# Ensuring continued progress in biomarkers for amyotrophic lateral sclerosis

**DOI:** 10.1002/mus.24470

**Published:** 2014-11-24

**Authors:** Martin R Turner, Michael Benatar

**Affiliations:** 1 Oxford University, Nuffield Department of Clinical Neurosciences, John Radcliffe HospitalWest Wing Level 3, Oxford, OX3 9DU, UK; 2 Miller School of Medicine, University of MiamiMiami, Florida, USA

**Keywords:** biomarker, neurochemistry, neuroimaging, motor neuron disease, trial

## Abstract

Multiple candidate biomarkers for amyotrophic lateral sclerosis (ALS) have emerged across a range of platforms. Replication of results, however, has been absent in all but a few cases, and the range of control samples has been limited. If progress toward clinical translation is to continue, the specific biomarker needs of ALS, which differ from those of other neurodegenerative disorders, as well as the challenges inherent to longitudinal ALS biomarker cohorts, must be understood. Appropriate application of multimodal approaches, international collaboration, presymptomatic studies, and biomarker integration into future therapeutic trials are among the essential priorities going forward.

In the last 5 years, the term *biomarker* has moved up the list of active research fronts in the fight to improve the still desperate outlook for those who face the adult-onset neurodegenerative disorder amyotrophic lateral sclerosis (ALS).^1^ Nearly a quarter of a century ago, in a provocatively entitled review, “Biological markers in ALS: help or hindrance?” Bradley recognized the obstacles of our incomplete understanding of basic disease mechanisms, the significant heterogeneity in survival, and the unacceptable size and length of therapeutic trials in ALS. He concluded that biological markers “…are essential components in our struggle both to discover the cause of ALS and to find a cure.”^2^

There has been progress. Since the first biomarker study in ALS in 1965,^3^ multiple candidate biomarkers have emerged,^4^ specifically from the neurochemical analysis of biofluids (cerebrospinal fluid, serum, and urine),^5^ advanced neuroimaging (positron emission tomography, magnetic resonance imaging),^6^ and neurophysiological techniques, including electromyography (EMG), transcranial magnetic stimulation,^7^ and electrical impedance myography.^8^ However, relatively few of these studies have been replicated, and only EMG (with a sensitivity of 57% and a specificity of 97%^9^) has become part of routine clinical practice. The ALS biomarker field is arguably still what Poste referred to generically as a “…dismal patchwork of fragmented research.”^10^ Can the wheels be kept from falling off the ALS biomarker bandwagon, and is its preservation still a worthy enterprise?

## BIOMARKER TYPES

The term *biomarker* encompasses a number of different but related concepts. The United States Food and Drug Administration recognizes diagnostic, prognostic, predictive, and pharmacodynamic biomarkers, to which we would add the category of disease-progression biomarkers (Table1). The purpose of a diagnostic biomarker is to identify patients with ALS and to differentiate them from patients with disease mimics that may otherwise be mistaken for ALS. Prognostic biomarkers, which help to categorize patients by the degree of risk for disease occurrence or progression, differ from predictive biomarkers, which categorize patients by their likelihood of response to a particular treatment. By contrast, biomarkers of disease progression are characteristics that are measurable and which change over time, and pharmacodynamic biomarkers are those for which a change in the biomarker indicates the occurrence of a biological response after a therapeutic intervention.

**Table 1:** Different categories of biomarker and their relationship to single or multiple measurements and clinical evaluations.

	Single clinical evaluation	Multiple clinical evaluations	
		Natural history study	Therapeutic intervention
Biomarker measured on a single occasion	Diagnostic	Prognostic	Predictive
Biomarker measured on multiple occasions	NA	Disease progression	Pharmacodynamic

## LIMITATIONS OF DIAGNOSTIC BIOMARKERS FOR ALS

The unmet biomarker needs in ALS differ significantly from those of other neurodegenerative diseases. Alzheimer disease is often preceded by a period of mild cognitive impairment, and rapid eye movement (REM) sleep behavior disorder may herald the future appearance of Parkinson disease. Disorders such as these may benefit from the development of accurate diagnostic biomarkers insofar as these prodromal clinical characteristics identify a discrete population in which a suitable biomarker could facilitate earlier diagnosis and therapeutic intervention. ALS, however, is not predictable for most patients. Screening strategies that target the general population are unlikely to be practical or feasible given the rarity of ALS in the general population (the estimated annual incidence is only ∼2 per 100,000 per year). There is no simple genetic screening test for the 90% of apparently sporadic cases, and there are no known large-effect premorbid risk factors.

The latency from symptom onset to diagnosis of ALS is unacceptably long (on average, 12 months^11^). However, the delay largely reflects the time it takes for the realization that referral to a neurologist is required. A firm diagnosis of ALS relies on the demonstration of both upper motor neuron (UMN) and lower motor neuron (LMN) involvement, and the former are frequently occult clinically.^12^ More slowly progressive cases of ALS are dominated by those with either clinically LMN-only forms of ALS (variably termed progressive muscular atrophy), or the much rarer pure UMN-only cases (termed primary lateral sclerosis).^13^,^14^ These slow progressors are also the individuals with the greatest diagnostic delay,^15^ who frequently miss out on therapeutic trials as a result. Although true mimics of ALS are rare in clinical practice,^16^ patients with LMN-only disease are a frequent challenge.^12^ Postmortem studies reveal that many of these individuals have occult UMN involvement,^17^,^18^ so that biomarkers of this UMN pathology would have clear diagnostic value in facilitating earlier access to therapeutic trials.

Notwithstanding the lack of a diagnostic test, neurologists expert in ALS have little difficulty reaching a confident diagnosis based on the medical history, physical examination, and, when necessary in those with limited clinical evidence of LMN involvement, the results of EMG. Despite the apparent disparate nature of the genes implicated in hereditary forms of ALS, the clinical syndrome common to all cases, hereditary or not, is typically one that no neurologist would fail to recognize. Meanwhile, just as important an approach to shortening the diagnostic latency may be targeted education of primary care physicians and the general population about “painless, progressive weakness or dysarthria” in an effort to reduce inappropriate referral or operative intervention.^19^

## PROGNOSTIC, PROGRESSION, AND PHARMACODYNAMIC BIOMARKERS

Therapeutic development efforts in ALS stand to benefit enormously from the development of prognostic, disease progression, and pharmacodynamic biomarkers. Biomarkers that predict prognosis could be used to stratify patients entering clinical trials at the time of randomization, thereby eliminating a major source of phenotypic heterogeneity that may be confounding current therapy development. Biomarkers of disease progression would find important application in the middle stages (i.e., phase II) of drug development. There is currently reliance on clinical endpoints to select drugs worthy of further evaluation in large phase III clinical trials. Because these clinical endpoints typically require prolonged follow-up (typically 9–18 months), phase II studies frequently take the form of underpowered phase III trials, and dose ranging is not accomplished effectively. Reliable and robust biomarkers of disease progression that are measurable over the short term (i.e., 3–4 months) would help to overcome these challenges. Biomarkers that demonstrate the target engagement of an experimental compound or, better still, the intended biological effect of the drug, would similarly impact the success of phase II clinical trials significantly.

## LIMITATIONS OF ALS BIOMARKER DISCOVERY COHORTS

Because there is invariably a diagnostic delay between symptom onset and diagnosis, ALS patients who are enrolled in longitudinal biomarker studies are inevitably evaluated for the first time at some point *after* the initial appearance of symptoms. Such studies, therefore, almost never capture biomarker data at symptom onset (and certainly not at disease onset). Moreover, there is an inverse correlation between the symptom onset to diagnosis latency and the rate of disease progression (shorter latency being associated with a more aggressive disease course^15^). This means that longitudinal biomarker discovery cohorts and therapeutic trial populations tend to enrich (i.e., select) for patients with “atypical,” more slowly progressive disease, and those with aggressive disease are less likely to be available for any longitudinal biomarker sampling. A similar phenomenon has been noted in relation to the symptom of cognitive impairment in ALS, whereby longitudinal studies enrich for those without significant involvement, by virtue of it being a surrogate marker of more aggressive disease.^20^

## WHAT NEEDS TO HAPPEN?

### The 3 Rs: Replicate, Replicate, Replicate

“The first principle is that you must not fool yourself.” The physicist and Nobel Laureate Richard Feynman recognized the fundamental value of replicating the published experiment as the initial step.^21^ This idea embodies the critical distinction between “discovery” and “validation” and the essential need for the latter. Although funding agencies and scientific journals may place less value on replication, open-access repositories for sharing neurochemical biomarker data (particularly from negative studies) would be a valuable development, along the lines of the ALS online genetics database (e.g., http://alsod.iop.kcl.ac.uk/Index.aspx).

### Longitudinal Studies

Despite the bias of enrichment for slower phenotypes, longitudinal studies should be the gold standard for biomarker validation. As part of this drive, a biomarker development arm should be a mandatory component in all future therapeutic trials.^22^ Essential to all biomarker development efforts, and certainly all longitudinal studies, is the need to adjust for sources of heterogeneity in interpreting results. Patients enrolled from specialist clinics into biomarker studies are typically quite heterogeneous with respect to duration of disease and degree of disability. Unlike other neurodegenerative disorders, the timing of symptom onset in ALS can usually be accurately defined, with the initial clinical presentation being focal limb (∼65%), bulbar (25%), cognitive (5%), or respiratory system (<5%) impairment. As a result ALS biomarker studies benefit from a consistent baseline by which to standardize patients (and thereby to reduce a source of heterogeneity). Progress in developing systems for staging disease^23^–^25^ add similar value insofar as they provide a mechanism for grouping together patients who have progressed to similar extents. More broadly, reanalysis of historical data sets in light of dates of death might allows biomarker sampling points to be stratified according to a more standardized timeline along the course of disease. These approaches must also incorporate the variability in rate of disease progression, which may be the most biologically relevant parameter. The current benchmark for prognostication is the rate in decline of the revised ALS Functional Rating Score—Revised (ALSFRS-R).^26^

### Multivariate Modeling

It seems improbable that a single biomarker would capture fully the complex biology of ALS. Instead, an array of clinical, neurophysiological, neuroimaging, and neurochemical biomarkers may provide an accurate individualized disease signature. Indeed, this is an accurate description of the biomarker landscape in Alzheimer disease.^27^ Multivariate approaches have been attempted using neurochemical markers in ALS and appear to offer potential for prognosis prediction.^28^ Such sophisticated multivariate modeling is particularly challenging to apply to repeated-measures data however.

Machine-learning algorithms may have value for identification of features that might not meet the statistical thresholds used in more standard multiple comparisons or in identification of patterns through iterative “fitting” approaches that would not be possible to undertake manually. These approaches have been used to identify latent prognostic clusters in ALS^29^ and diagnostic classifiers from neuroimaging data.^30^,^31^ A “big data” solution for ALS, whether at the genetic, proteomic, metabolomics, or functional magnetic resonance imaging (MRI) connectivity systems level, may not necessarily deliver a “Unified Theory of ALS,” despite ever finer granularity of clinical, genetic, and molecular phenotyping. Nonetheless, the ability to stratify patients more confidently early in the disease course using a multiple biomarker “signature” approach would have clear benefit.

Equally, the very high statistical thresholds required for the inherent multiple comparisons that occur in large data sets may silence some important biomarker signals and, undoubtedly, the complexity of biological systems continues to be underestimated. Both issues mean it will be essential to invest in the bioinformatics expertise required to implement and interpret such approaches.

### Standardization of Techniques and Data-Sharing

Poste emphasized that none of the advantages of the big data approaches can be realized without standardization of sample collection.^10^ It is becoming clear that variability of collection, storage, and analysis within and across laboratories is a major barrier to clinical translation of emerging biomarkers. Although potential sources of variability in samples are daunting,^32^,^33^ international collaborative initiatives toward standardization and harmonization (e.g., http://www.sophiaproject.eu/) have been recognized as important, with roadmaps emerging for neurochemical^34^ and neuroimaging biomarkers.^35^ Data-sharing across international boundaries will be an important aspect of future success, and academic-led initiatives, such as the Neuroimaging Society in ALS (www.nisals.org), have demonstrated that this is feasible.

### Use of Relevant Controls

Although diagnostic biomarkers are not the research priority for ALS (discussed above), the literature focused on development of this sort of biomarker provides an instructive example of the importance of selection of appropriate controls. The question a neurologist asks when faced with a potential ALS patient is not, by and large, whether the individual is healthy, yet the vast majority of candidate-generating biomarker discovery studies have been conducted with direct comparison of patients and healthy age-matched controls. Even when diagnostic biomarker discovery studies include ALS disease mimics, these studies invariably employ a “case–control” design in which people known to have ALS are compared with those known to not have ALS. This too is artificial insofar as this is not the population in which the diagnostic biomarker would be used clinically. It would be more robust to utilize a “cohort” design, in which consecutive patients suspected (but not yet proven) to have ALS are evaluated using the putative biomarker. Over time, some of these people will go on to develop ALS and others will not (i.e., the true disease mimics). Available data indicate that the “case–control” approach tends to overestimate diagnostic accuracy by a factor of 2–3.^36^ The development of UMN biomarkers has significant potential to reduce diagnostic delay in clinically LMN-only cases, and the incorporation of LMN disease controls (e.g., multifocal motor neuropathy) will be a major part of their development. Finally, intragroup comparison of fast versus slow progressors is another potentially useful way to identify biomarkers of greatest relevance to the fundamental biological processes in ALS. Such contrasts arguably also control for a wider range of factors than healthy population comparisons.

### Presymptomatic Studies

Biomarker studies in the presymptomatic period, currently only feasible for those at genetic risk for developing ALS, are essential for several reasons.^37^ First, they empower the study of both disease onset and symptom onset (overcoming biases inherent in studies that focus exclusively on the already affected population). Second, because the presymptomatic phase eventually evolves into symptomatic disease, such studies offer the potential to study even those with the most aggressive forms of the disease (because participants are being followed from the earliest possible time-point). This mitigates the bias toward those with more slowly progressive forms of disease when biomarker studies enroll only those who already have manifest disease. However, an important caveat is that the results derived from studies involving presymptomatic relatives of the ∼10% of ALS patients linked to a single genetic mutation, may differ qualitatively from those with non-genetic forms of disease, and thus it may be difficult to extrapolate all findings to the majority with apparently sporadic disease.

Furthermore, testing of the only disease-modifying therapy in ALS, riluzole, in relation to potentially pharmacodynamic biomarkers in this setting is an important new front in the battle to develop new therapies, albeit a much higher bar in terms of development. Such studies can provide proof of target engagement and offer a first glimpse of a future of primary prevention for some patients.

## CONCLUSIONS

Biomarker development is still an essential component of future therapeutic development in ALS. It is gaining momentum on a new wave of biomathematical approaches, coupled with wider international collaborative efforts. Some of the challenges inherent to the way ALS presents to healthcare professionals have become clearer, and more will certainly emerge. It is also important to acknowledge the value of biomarker research in supporting or revealing fundamental pathophysiological mechanisms in ALS,^38^–^40^ as well as for the goals of improved stratification and therapeutic monitoring.

The biomarker endeavor in ALS encapsulates a wider landmark in Medicine, one in which the Oslerian clinical approach to disease classification is colliding increasingly with a big data–driven approach. It is their eventual integration, rather than a superseding of one by the other, that will lead to new heights in tackling the global catastrophe of neurodegenerative disease.

## Abbreviations

ALS: amyotrophic lateral sclerosis
ALSFRS-R: revised ALS Functional Rating Score
EMG: electromyography
LMN: lower motor neuron
NiSALS: Neuroimaging Society in ALS
REM: rapid eye movement
SOPHIA: Standardization, Optimization and Harmonization of Biomarkers in ALS
UMN: upper motor neuron
